# *HTRA1-AS1*, an *ARMS2*-region long non-coding RNA, is downregulated in retinas of age-related macular degeneration patients

**DOI:** 10.1101/2025.10.29.25338834

**Published:** 2025-11-06

**Authors:** Ping-Wu Zhang, Zi-He Wan, Sheng Liu, Jie Wang, Srinivasa Sripathi, Weifeng Li, Junyeop Ahn, Sean Li, Laura Fan, Cynthia A. Berlinicke, Jiang Qian, Shannath L. Merbs, Donald J. Zack

**Affiliations:** 1. Department of Ophthalmology, Stem Cell Ocular Regenerative Medicine Center, Wilmer Eye Institute, Johns Hopkins University School of Medicine; Baltimore, MD, 21231, USA; 2. Department of Medical and Molecular Genetics, Center for Computational Biology and Bioinformatics, Indiana University School of Medicine, Indianapolis, IN, 46202, USA; 3. Henderson Ocular Stem Cell Laboratory, Retina Foundation of the Southwest, Dallas, 75231, USA; 4. Department of Ophthalmology, University of Virginia School of Medicine, Charlottesville, VA, 22908, USA; 5. Solomon H. Snyder Department of Neuroscience, Department of Molecular Biology and Genetics, Department of Genetic Medicine, Center for Nanomedicine at the Wilmer Eye Institute, The Johns Hopkins University School of Medicine, Baltimore, Maryland, United States

**Keywords:** Age-related macular degeneration (AMD), long non-coding RNA (lncRNA), *HTRA1-AS1*, *ARMS2*, *HTRA1*, single-nucleotide polymorphism (SNP), human retina, cigarette smoke extract (CSE), induced pluripotent stem cells (iPSC), retinal pigment epithelium (PRE), paraquat

## Abstract

**Purpose:**

The human 10q26 locus is a major genetic risk factor for age-related macular degeneration (AMD). Fine mapping by linkage and large-scale genome-wide association studies (GWAS) has narrowed this region to a 30-kb interval encompassing the *ARMS2* and *HTRA1* genes. However, the causative gene(s), risk variants, and underlying pathogenic mechanisms remain unresolved.

**Methods:**

Long non-coding RNA (lncRNA) candidates within the *ARMS2–HTRA1* region were identified using human postmortem retinal RNA-seq data and public databases (NCBI, Ensembl). Candidate transcripts were validated by RT-PCR and Sanger sequencing. Published single-cell RNA-seq datasets were analysed to define cell type–specific expression, and RNA levels were compared between AMD and non-AMD donor retinas. Additionally, expression changes were assessed in human iPSC-derived retinal pigment epithelium (RPE) cells exposed to cigarette smoke extract (CSE) and paraquat (PQT).

**Results:**

We identified and validated a lncRNA, *HTRA1-AS1*, and its transcript variants (*ENST00000647969.1*) within the *ARMS2* locus. *HTRA1-AS1* overlaps *ARMS2* and is transcribed in the antisense orientation. It is predominantly expressed in rod photoreceptors, Müller glia and Choroid/RPE, and its retinal expression was significantly reduced in AMD compared with controls (43 AMD donors vs. 44 controls, p = 0.007). By contrast, *HTRA1* mRNA showed no significant difference (p = 0.121). Furthermore, *ENST00000647969.1, HTRA1-AS1* and *ARMS2* expression increased dramatically, up to 101-fold, 8-fold and 75-fold, respectively, in induced pluripotent stem cells (iPSC)-derived RPE cells following cigarette smoke extract (CSE)-induced oxidative stress but showed no significant change after paraquat treatment.

**Conclusion:**

These findings suggest that *HTRA1-AS1*, a dysregulated lncRNA within the *ARMS2* locus, may act as a non-coding element contributing to transcriptional mis-regulation underlying AMD pathogenesis.

## Introduction

Age-related macular degeneration (AMD), the leading cause of irreversible blindness in the elderly, is characterized by the progressive degeneration of photoreceptors and retinal pigment epithelial (RPE) cells.^1^ In 2019, the prevalence of early-stage AMD among adults aged 40 years and older in the United States was estimated at 11.64%.^2^ While aging, smoking, and diet constitute key environmental factors, numerous studies have established the importance of genetic contributions to AMD risk.^3^

Although approximately 70–80% of AMD cases are sporadic, genetic factors have been suggested to account for up to 70% of overall disease risk.^4–6^ This contribution is comparable to that observed in Alzheimer’s disease (AD) and significantly greater than in other common neurodegenerative disorders like amyotrophic lateral sclerosis (ALS) and Parkinson’s disease (PD). For comparison, 90–95% of ALS and PD cases are sporadic, and known pathogenic variants explain only 10–20% of ALS and ~14% of PD susceptibility.^7–10^ Conversely, in late-onset AD, APOE ϵ4 is the strongest risk factor, and genome-wide association study (GWAS) identified variants collectively explain ~70% of disease susceptibility.^11, 12^

AMD risk factors are complex and multigenetic. To date, more than 40 genes and loci have been associated with AMD, with the complement factor H (*CFH*) locus on chromosome 1q31 and the *ARMS2/HTRA1* locus on chromosome 10q26 showing the strongest associations.^6, 13^ Together, these two loci account for more than 50% of the inherited risk for AMD.^14^ The remaining genetic risk is distributed across a variety of more minor loci that together account for about 20% of heritability.^4, 6^ Ethnic variability has been observed for *CFH* variants, which show strong associations in Caucasian populations but inconsistent replication in some east Asian cohorts.^15–17^ In contrast, the *ARMS2/HTRA1* locus has demonstrated robust associations across diverse populations, with no clear inter-ethnic differences. Furthermore, rod-mediated dark adaptation (RMDA) - a functional biomarker of early AMD - has been consistently linked to *ARMS2*, but not *CFH* risk alleles, even in individuals without overt disease.^18^

At the functional level, the causal variation at the *CFH* locus is relatively well defined, implicating its role as a central regulator of the complement pathway. By contrast, the precise biological contribution of the *ARMS2/HTRA1* locus to AMD remains incompletely understood. *HTRA1* encodes a serine protease, but the function of *ARMS2* is still unclear, and the specific gene(s), risk variants, and molecular mechanisms driving AMD susceptibility at 10q26 are not yet established.^5, 19^

Given that the *ARMS2/HTRA1* locus remains one of the most strongly associated yet mechanistically least understood regions in AMD genetics, we sought to perform an in-depth investigation of this genetic interval. Our study aimed to identify novel coding or noncoding transcripts within the *ARMS2/HTRA1* region that may contribute to AMD pathogenesis and provide mechanistic insight into how this locus confers risk.

### Ethics statement

All aspects of this study were conducted in accordance with the principles of the Declaration of Helsinki, with informed consent being obtained from all participants. This project was approved by the Johns Hopkins University School of Medicine Institutional Review Board (IRB).

## Material and Methods

### Human AMD eye samples

Post-mortem eye tissues (AMD and non-AMD controls) were obtained from the National Disease Research Interchange (NDRI, Philadelphia, USA) and transported on wet ice. Processing of all samples occurred within 48 hours of death. Before retina isolation, macular photography was performed to document pathology. AMD samples were included only if they had documented clinical history and gross macular pathology consistent with disease. All tissue handling and processing procedures complied with the ARVO Best Practices for Using Human Eye Tissue in Research (Nov 2021).

### RNA Extraction from human retinal tissues

Total RNA was extracted from ~50 mg of retinal tissue using TRIzol (Thermo Fisher Scientific, Invitrogen, USA) and purified with the PureLink RNA Mini Kit (Thermo Fisher Scientific, Invitrogen, USA), including on-column DNase digestion (Thermo Fisher Scientific, Invitrogen, USA). Following homogenization in 600 μL TRIzol, samples underwent chloroform extraction and ethanol precipitation. RNA was purified using spin cartridges (Thermo Fisher Scientific, Invitrogen, USA) and eluted in RNase-free water. RNA concentration and purity were measured on a NanoDrop spectrophotometer (Thermo Fisher Scientific, USA), and samples with A260/280 ratios between 1.7 and 2.1 were used for downstream experiments.

### Human iPSC Culture and RPE Differentiation

Human-induced pluripotent stem cells hiPSCs; EP1 line were cultured and differentiated into RPE monolayers following established protocols.^20^ Briefly, hiPSCs were maintained on growth factor–reduced Matrigel (BD Biosciences) in mTeSR1 medium (Stem Cell Technologies) under 10% CO_2_ and 5% O_2_, with expansion promoted by 5 μM blebbistatin (MilliporeSigma). For differentiation, cells were seeded at 35,000 cells/cm^2^ in mTeSR1 until confluency, then switched to differentiation medium (DMEM/F12 supplemented with 15% knockout serum, 2 mM glutamine, 1% nonessential amino acids, 0.1 mM mercaptoethanol, 1% antibiotic–antimycotic, and 10 mM nicotinamide, all are from Thermo Fisher Scientific, USA) for ~50 days. Differentiating RPE cells were dissociated with 0.25% collagenase IV (Thermo Fisher Scientific, USA) and AccuMAX (MilliporeSigma, USA) to generate a single-cell suspension, then re-plated on Matrigel-coated plates. Cells were matured for 2–3 months in RPE medium (70% DMEM, 30% Ham’s F12, 2% B-27 supplement, and 1% antibiotic–antimycotic, all were from Thermo Fisher Scientific, USA).

### Cigarette Smoke Extract (CSE) and paraquat treatments for iPSC-derived RPE monolayers

Human iPSC-derived RPE monolayers were differentiated using our established methods and cultured for eight weeks to achieve mature RPE cultures.^21^ The mature monolayers were then treated for 48 hours with Cigarette Smoke Extract (CSE) (Murty Pharmaceuticals, USA) and paraquat (MilliporeSigma, USA) at three lethal dose (LD) concentrations: LD25, LD50, and LD75 (doses for CSE: 125, 250, and 500 μg/mL, respectively; doses for PQT: 100, 300, and 600 μM, respectively). The CSE was prepared in DMSO, with the final solution containing 40mg/mL condensate. Following treatment, total RNA was extracted from the RPE monolayers using RNeasy Mini Kit (QIAGEN, USA). RNA purity and concentration were assessed using NanoDrop^™^ spectrophotometry (Thermo Fisher Scientific, USA), and only samples with A260/A280 ratios between 1.8 and 2.1 were used for downstream qPCR analysis.

### Reverse transcription and Quantitative PCR (qPCR)

Total RNA was reverse-transcribed into cDNA using either the High-Capacity cDNA Reverse Transcription Kit (Applied Biosystems, USA) or the iScript^™^ cDNA Synthesis Kit (Bio-Rad, USA). Quantitative PCR (qPCR) was then performed on a CFX-384 Real-Time PCR system using iTaq Universal SYBR Green Supermix (Bio-Rad, USA). All reactions were conducted in biological triplicates. Relative gene expressions were calculated using the geometric mean of two housekeeping genes, GAPDH and 18S (use a 100-fold dilution of 18S cDNA for qPCR analysis), for normalization. Primer sequences are detailed in Supplementary Table S1.

### RNA-sequencing (RNA-seq) and data analysis

Published RNA-seq datasets were re-analysed for expression of genes within the *PLEKHA1–ARMS2–HTRA1* locus.^22^ Reads were mapped to the region of interest and quantified using standard pipelines.

### Sanger Sequencing

DNA sequencing was performed at the Johns Hopkins Genetics Resources Core Facility using the Applied Biosystems 3730xl DNA Analyzer (Thermo Fisher Scientific, USA).

### Software and Statistical Analysis

Data were analysed using GraphPad Prism (GraphPad Software10.3, USA) and Microsoft Excel. Statistical significance was assessed with unpaired two-tailed Student’s t-tests unless otherwise indicated. Data are presented as mean ± SEM (n = biological replicates). ns = not significant; * p < 0.05; ** p < 0.01; *** p < 0.001; **** p < 0.0001. A p-value < 0.05 was considered statistically significant. AlphaFold3 was used for predicting the three-dimensional structures of the putative peptide coding regions.

## Results

### RNA-seq identifies aligned-read peaks overlapping *HTRA1-AS1*

**Figure 1.**
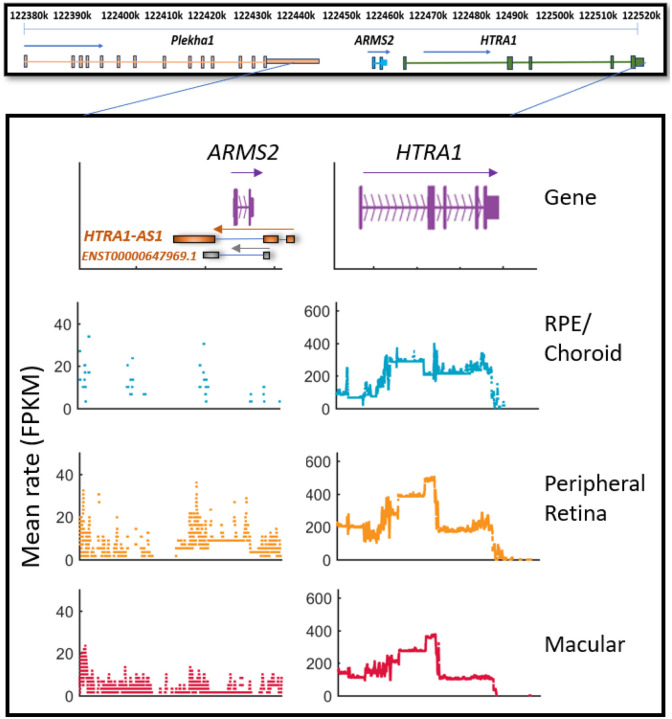
RNA-seq expression of 10q26 locus genes in human ocular tissues. Genomic organization of *PLEKHA1*, *ARMS2*, and *HTRA1* is shown, with exons represented by thick boxes and introns by lines. The antisense transcript *HTRA1-AS1* and *ENST00000647969.1* are also shown in orange, overlapping the *ARMS2* and *HTRA1* loci. Additional RNA aligned-reads peaks were detected in the *ARMS2* region and overlap with lncRNA *HTRA1-AS1* gene and another transcript variant *ENST00000647969.1*.

Reanalysis of RNA-seq datasets from macular retina, peripheral retina, and RPE/choroid tissues across the *ARMS2–HTRA1* locus revealed two prominent read-alignment peaks upstream and one downstream of *ARMS2*, consistently detected in both retinal and RPE/choroid samples (Figure 1). *HTRA1* exhibited robust expression across all ocular tissues (100–550 FPKM) with no marked preference between retina and RPE, whereas *ARMS2* expression was minimal and largely confined to the retina. The antisense transcript *HTRA1-AS1* (annotated on NCBI as predicted *HTRA1* and *ARMS2* antisense RNA 1), together with *ENST00000647969.1*, were expressed at modest levels (~30–50 FPKM), representing only a small fraction of *HTRA1* expression. These antisense peaks correspond to low-abundance noncoding transcription within the 10q26 risk interval, supporting the presence of additional regulatory elements in this region.

### Validation of lncRNA *HTRA1-AS1* and transcript variants

**Figure 2.**
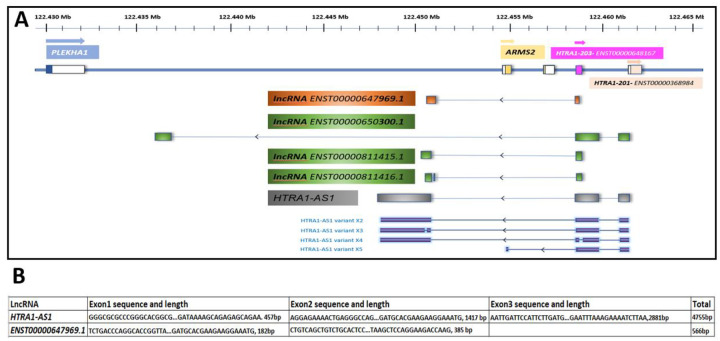
Schematic portraying the overlap of the association peak with the ARMS2 gene and lncRNA variants overlap with ARMS2 gene. **(A)** LncRNA candidates and their locations. Genomic map of the *PLEKHA1*–*ARMS2*–*HTRA1* region on chromosome 10q26, illustrating annotated protein-coding genes (blue, yellow and coral), and several lncRNAs (shaded in green, orange, and grey). Coordinates are given in mega base (Mb). *HTRA1-AS1* (grey) and predicted four transcript variants (X2–X5, shown in dark blue) are located antisense to the *ARMS2* gene. Several other lncRNAs (*ENST00000647969.1*, *ENST00000650300.1, ENST00000681145*.1, and *ENST00000681146.1*) are positioned within or near the *ARMS2* locus, suggesting potential regulatory roles. Exons are indicated by boxes and introns by connecting lines. Direction of transcription is shown by arrows. (**B)** Boundary sequences of exons for lncRNA *ENST00000647969.1* and *HTRA1-AS1*.

Database searches identified multiple long non-coding RNAs (lncRNAs) overlapping *ARMS2*. Ensembl listed four candidates (*ENST00000650300.1, ENST00000647969.1, ENST00000811415.1*, and *ENST00000811416.1*), and NCBI annotated *HTRA1-AS1* (*LOC105378525*) with four predicted transcript variants (XR_946382–XR_946385). All were transcribed antisense to *ARMS2* (Figure 2A).

RT-PCR and Sanger sequencing confirmed expression and boundary sequences of *HTRA1-AS1* and *ENST00000647969.1* in human retinas (Figure 2B). *HTRA1-AS1* contains three exons (457 bp, 1417 bp, and 2881 bp, total 4755 bp) with sequence segments provided.

*ENST00000647969.1* includes two exons (182 bp, 385 bp, total 566 bp). Other predicted variants were weakly or not detected. *ENST00000647969.1* appears to represent an alternative splice isoform of *HTRA1-AS1*.

### *HTRA1-AS1* is downregulated in human AMD retinas

**Figure 3.**
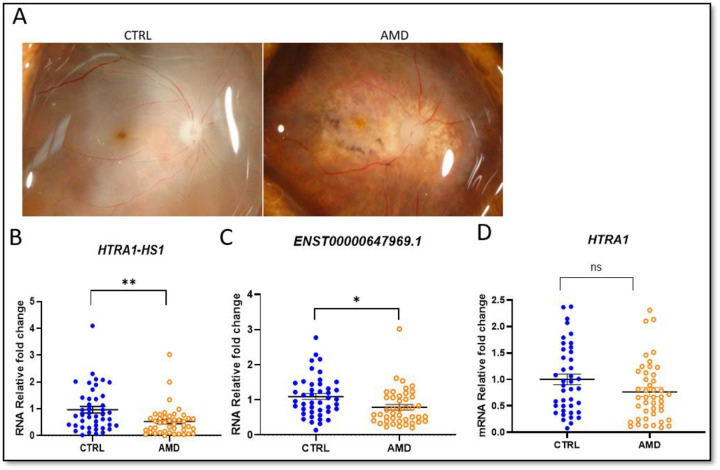
RT-qPCR analysis of RNA expression in control and AMD donor retinas. (A) Representative macular images from control and AMD eyes. (B) *HTRA1-AS1* expression is reduced in AMD (p < 0.01). (C) Transcript *ENST00000647969.1* is decreased in AMD (p < 0.05). (D) *HTRA1* expression shows no significant difference (p > 0.05).

To assess the relationship between lncRNA *HTRA1-AS1* expression and age-related macular degeneration (AMD), we collected human AMD and non-AMD control donor eyes from the National Disease Research Interchange (NDRI) over a period of more than five years. Donor demographic and clinical characteristics are summarized in Table 1. Most donors were White, and the two groups were age- and sex-matched. The mean donor age was 81.4 years in the control group and 82.5 years in the AMD group. Sex distribution was also comparable (control: 19 males, 24 females; AMD: 22 males, 21 females). Representative images of control and AMD eyes are shown in Figure 3A.

*HTRA1-AS1* and *ENST00000647969.1* were significantly downregulated in AMD eyes (p = 0.007 and p = 0.041, respectively; Figure 3B, 3C). In contrast, *HTRA1* mRNA levels did not differ significantly between AMD and controls (p = 0.121; Figure 3D). *ARMS2* expression was extremely low (Ct >34) in our samples and not reliably quantifiable.

### Cell-type expression profiles of *HTRA1-AS1* and *HTRA1*

**Figure 4.**
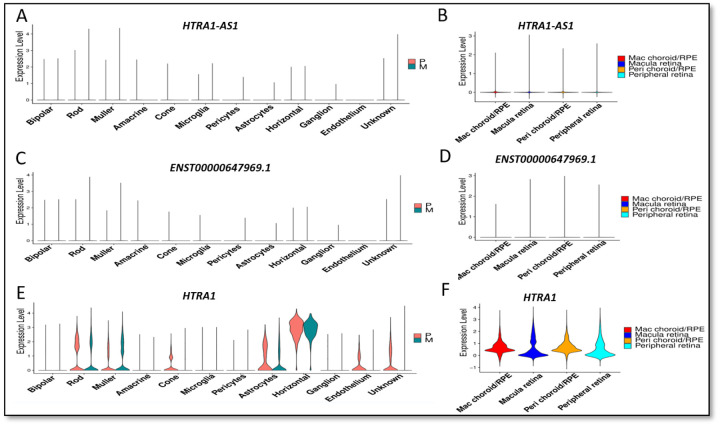
Single-cell expression profiles of lncRNAs and HTRA1 across ocular cell types and tissue regions. Violin plots show the expression distributions of *HTRA1-AS1*, *ENST00000647969.1*, and *HTRA1* across various ocular cell types and tissue compartments derived from human donor eyes. Expression levels are normalized and plotted per cell type or tissue region. (A) *HTRA1-AS1*(C) ENST00000647969(E) *HTRA1*. Color coding indicates cells from macula (M) and peripheral (P) regions. (**B, D, F**) Regional expression of the same genes across four tissue compartments

To characterize the cell-type and regional expression patterns of *HTRA1-AS1*, *HTRA1*, and transcript *ENST00000647969.1*, we analyzed publicly available single-cell RNA sequencing datasets from human retina and RPE/choroid tissues. *HTRA1-AS1* exhibited cell-type specific expression, with prominent enrichment in photoreceptors, particularly rods and cones, as well as Müller glia and RPE/choroid (Figure 4A, 4B). Similarly, transcript *ENST00000647969.1* showed a comparable expression pattern to *HTRA1-AS1*, with enrichment in photoreceptors and Müller glia, but low regional expression in both macular and peripheral tissues (Figures 4C and 4D). In contrast, *HTRA1* displayed a broader expression profile, with detectable levels across multiple retinal cell types including photoreceptors, astrocytes, and microglia (Figure 4E). Notably, *HTRA1* was robustly expressed across all retinal and RPE/choroid regions, with comparable expression levels in both macula and peripheral tissues (Figure 4F). These data suggest that while *HTRA1* is widely expressed across retinal compartments, *HTRA1-AS1* and ENST00000647969 exhibit more cell-type restricted expression patterns with limited regional variation, pointing to potentially distinct regulatory roles in specific retinal cell populations.

Available data indicate that *HTRA1-AS1* and *ENST00000647969.1* are primarily expressed in rod photoreceptors, Müller glia and Choroid/RPE, whereas *HTRA1* is enriched in horizontal cells and moderately expressed in rods, cones, astrocytes, Müller glia, and RPE/choroid. Minimal or no *HTRA1* expression was detected in microglia, bipolar, ganglion, or Amacrine cells. *HTRA1* mRNA expression was notably higher in RPE/choroid compared to retina in both peripheral and macular regions. However, *HTRA1-AS1* and *ENST00000647969.1* did not show significant cellular differences in expression between the retina and RPE/choroid (Figure 4A–F).

### Cigarette smoke extract (CSE), but not paraquat (PQT), selectively induces dose-dependent upregulation of *HTRA1-AS1* and *ARMS2* mRNA.

**Figure 5.**
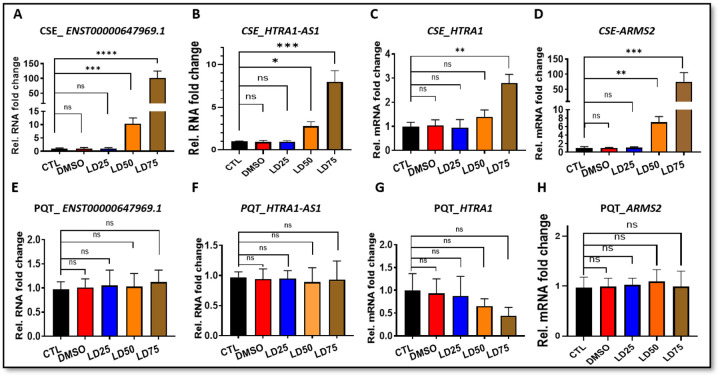
Quantitative RT-PCR analysis of the expression of *ENST00000647969.1, HTRA1-AS1, HTRA1*, and *ARMS2* mRNA in cells following treatment with increasing concentrations of CSE (Panels A–D) or PQT (Panels E–H). Treatment groups included untreated control (CTL), vehicle control (DMSO), and three lethal dose (LD) levels: LD25, LD50, and LD75. mRNA levels were normalized to GAPDH and 18S. (A–D).

Because cigarette smoking is a major risk factor for AMD, we investigated the transcriptional response of *HTRA1-AS1* and neighbouring genes to CSE in iPSC-derived RPE cells. CSE treatment elicited a robust, dose-dependent induction of *ENST00000647969.1*, *HTRA1-AS1*, and *ARMS2* transcripts, with the highest increases observed at the LD75 dose (101.4-fold, 8.0-fold, and 75.3-fold, respectively; Figure 5A, 5B, 5D). *HTRA1* mRNA expression also rose at higher CSE concentrations, though to a lesser extent, reaching a maximum 2.8-fold increase (Figure 5C).

To test if this response was specific to CSE or a general reaction to oxidative stress, we treated cells with paraquat (PQT), a classic oxidative stress inducer. In contrast, PQT treatment across comparable sublethal concentrations (LD25–LD75) did not significantly alter the expression of any of the tested transcripts (Figure 5E–H). These findings demonstrate that CSE selectively drives the dose-dependent induction of *HTRA1-AS1* and *ARMS2* in RPE cells, suggesting a smoking-specific regulatory effect on this AMD-associated locus.

## Discussion

A major challenge in AMD research is reconciling the strong genetic associations at the *ARMS2/HTRA1* locus with the limited molecular evidence supporting a causative mechanism. Previous investigations have largely focused on the protein-coding genes *ARMS2* and *HTRA1*.^23^ In the present study, we broadened this scope to noncoding transcripts within the 10q26 interval, which is consistently implicated by GWAS and transcriptomic data. Importantly, RNA read densities across this region often exceeded those mapped to *ARMS2* exons, suggesting that additional regulatory elements beyond *ARMS2* and *HTRA1* may contribute to disease risk. The identification of disease-associated genes typically relies on three strategies: candidate gene analysis, linkage studies with whole-exome or whole-genome sequence data in familial disease, and GWAS with targeted follow-up in sporadic disease. By integrating retinal RNA-seq with NCBI and Ensembl annotations, we identified a novel antisense lncRNA, *HTRA1-AS1*, (and its isoforms) that overlap with *ARMS2*.

Although several lncRNAs have been implicated in AMD,^24, 25^ none of the previously described lncRNAs are located within the *ARMS2/HTRA1* risk locus. A recent preprint reported the same lncRNA (*BX842242.1/ENSG00000285955*) associated with reticular pseudodrusen (RPD), with elevated expression correlating with reduced *HTRA1* levels.^26^ In contrast, our data demonstrate that *HTRA1-AS1* is significantly downregulated in AMD retinas, whereas *HTRA1* mRNA expression was unchanged between cases and controls. Such discrepancies likely reflect differences in tissue sources, retinal layers analyzed, or disease context, underscoring the complexity of regulatory networks within this locus.

Since the original 2006 discovery report for *ARMS2-HTRA1* region,^27^ attempts to directly link *ARMS2* or *HTRA1* expression with AMD have yielded inconsistent results, often due to limited sample sizes.^19^ By analyzing 43 AMD and 44 control retinas, our study represents the largest cohort to date demonstrating lncRNA dysregulation at this locus. Specifically, *HTRA1-AS1* was reduced in AMD retinas but was robustly induced by cigarette smoke extract (CSE) in iPSC-derived RPE cells. Interestingly, this effect was not observed with paraquat, a general oxidative stressor, indicating that *ARMS2* and *HTRA1-AS1* are not simply stress-inducible transcripts. Instead, their selective upregulation by CSE points to the involvement of specific, non-oxidative molecular cues derived from cigarette smoke. The modest but significant increase in *HTRA1* expression further supports a coordinated, gene-specific response. Together, these findings suggest that smoking may potentiate disease-associated transcriptional programs at the *ARMS2–HTRA1* locus, providing a direct mechanistic link between environmental and genetic risk factors in AMD.

**Figure 6.**
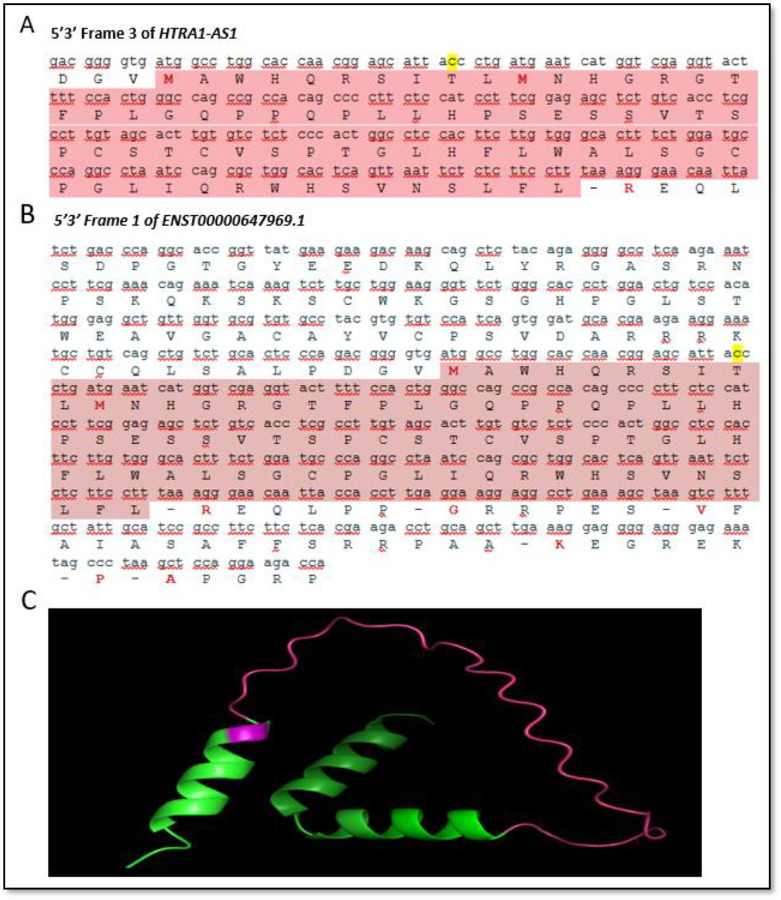
Predicted coding sequences and protein structure for HTRA1-AS1 and ENST00000647969.1. **A and B**, Nucleotide and corresponding amino acid sequences of the putative open reading frames (ORFs) identified in the 5′–3′ Frame 3 of *HTRA1-AS1* (**A)** and the 5′–3′ Frame 1 of *ENST00000647969.1* (**B**). Coding regions are shaded in pink, with the start codon (ATG) and stop codons highlighted in yellow. (**C**) Predicted three-dimensional structures of the peptides encoded by the putative ORFs in *HTRA1-AS1* and *ENST00000647969.1*, generated using AlphaFold. The structures display alpha-helices and loop regions, with the amino acid change caused by SNP rs61871745 indicated in pink.

The precise functional role of *HTRA1-AS1* remains to be elucidated. Although antisense lncRNAs frequently regulate the transcription or stability of neighboring genes,^23^ our data argue against a simple repressive interaction with *ARMS2*. Increasing evidence also indicates that some lncRNAs can produce bioactive micropeptides. For instance, the LIL peptide, derived from a two-amino-acid open reading frame, promotes cell growth.^28^ Notably, the AMD-associated SNP rs61871745 (G/A; A as the risk allele) is located within predicted coding regions of *HTRA1-AS1* and *ENST00000647969.1*. The risk allele A may induce a nucleotide substitution (C→T) that changes the predicted encoded codon from ACC to ATC (Thr→Ile) within one of the putative open reading frames.^29^ Structural modeling suggests that this amino acid substitution could play a role in the predicted peptide (Figure. 6). Whether *HTRA1-AS1* exerts its effects solely as a regulatory lncRNA, encodes a functional micropeptide, or possesses dual roles remains an intriguing question for future investigation.

In summary, our study identifies *HTRA1-AS1* and its isoform *ENST00000647969.1* as antisense lncRNAs overlapping ARMS2 and demonstrates their significant downregulation in AMD retinas. Their altered expression, stress responsiveness, and coding potential highlight new mechanistic pathways at the 10q26 locus. Pinpointing the causal variants and clarifying the potential regulatory and/or coding roles of *HTRA1-AS1* will be important to resolve the long-standing debate surrounding the *ARMS2/HTRA1* locus and may ultimately uncover new therapeutic opportunities for AMD.

## Supplementary Material

Supplement 1

## Data Availability

The authors declare that all data generated or analysed during this study are included in this published article.
