# Supplementary material for: HTRA1-AS1, an ARMS2-region long non-coding RNA, is downregulated in retinas of age-related macular degeneration patients
Source: medRxiv. 2025 Nov 6:2025.10.29.25338834. Preprint. [Version 1] doi: 10.1101/2025.10.29.25338834 (PMC12637771; doi:10.1101/2025.10.29.25338834)
Supplement: Supplement 1 [file media-1.pdf]

Supplementary data

1. Table S1 Primer sequences used for quantitative PCR (qPCR)

qPCR Primers	Primer sequences
hGAPDH-F	CTGACTTCAACAGCGACACC
hGAPDH-R	TAGCCAAATTCGTTGTCATACC
hENST00000647969-F	TCTACAGAGGGGCCTCAAGAA
hENST00000647969-R	CTGAAAGTCACATCAAGAATGGAA
hHTRA1-AS1-F	TCACCCCACCTGCTCTGACC
hHTRA1-AS1-R	ACAGCTGAAAGTCACATCAAGAATGGA
hHTRA1-F	AAGCCAAAGAGCTGAAGGACCG
hHTRA1-R	CTTCATTACCCCTGCGGACCAC
hARMS2-F	TGACAAGCAGAGGAGCAAACTGT
hARMS2-R	TGTTTTGGCAGAATCACCTTGCTG
ENST00000650300-qPCR-F1	CCTGGACTGTCCACATGGGA
ENST00000650300-qPCR-R1	CCATGTGGAGAAGCTTTGACC
ENST00000811415qPF2	ACATGGGAGGCTGTTGGTGC
ENST00000811415qPR2	GCTGGGCTTGGTCTTCCTGG
ENST00000811416qPF2	CACCAACGGAGCATTACCCTGA
ENST00000811416qPR2	CGGAATCATCACAGAGGCTGGG
h18SrRNA-F	CAGCCACCCGAGATTGAGCA
h18SrRNA-R	TAGTAGCGACGGGCGGTGTG

Note: Forward (F) and reverse (R) primers were designed for the detection of human *GAPDH* (housekeeping control), *ENST00000647969.1* (lncRNA isoform), *HTRA1-AS1* (lncRNA), *HTRA1*, and ARMS2 transcripts. All sequences are listed in the 5′ to 3′ direction.

2. The position of rs61871745 in long non-coding RNAs

SNP Rs61871745 **(original G/A, antisense** C/T) position is highlighted in yellow.

1) LncRNA *HTRA1-AS1 (LOC105378525=ENST00000285955)*

5'3' Frame 1

```
ggg cgc gcc cgg gca cgg cgg tgc tgg cgg ggc gcg ggc ggg gca gcg cac agg ccg gcc
 G   R   A   R   A   R   R   C   W   R   G   A   G   G   A   A   H   R   P   A
ctc tcg cgg gac tca gtt tcc ccg tct gcg cgc ccc aca tat tcg ctt tcc gca ggt tct
 L   S   R   D   S   V   S   P   S   A   R   P   T   Y   S   L   S   A   G   S
ctc gct gag att cgc gtc ctt caa act aat gga act ttg caa ggg ggc cct gcg gcg gct
 L   A   E   I   R   V   L   Q   T   N   G   T   L   Q   G   G   P   A   A   A
tcg cgt ggg agc gcc ggg gaa agt tcc tgc aaa tcg ccg gac tgg ggg ccc gcc cgg gaa
 S   R   G   S   A   G   E   S   S   C   K   S   P   D   W   G   P   A   R   E
gct cgg act ggg ccg ggc agg gac tgc agg ggc ctc tgc gca gac ccg ggg cgt tgc ggc
 A   R   T   G   P   G   R   D   C   R   G   L   C   A   D   P   G   R   C   G
acc gcg gac ccc ggt cac gcg ctg gtt ctg ccc ccg gta ccc cgc acg cgg gac cct gac
 T   A   D   P   G   H   A   L   V   L   P   P   V   P   R   T   R   D   P   D
cgc ggg gcc tct gcg gcc gga cga agg cag cgt ccg cgg agc tgg cag aaa gcc cgc gcc
 R   G   A   S   A   A   G   R   R   Q   R   P   R   S   W   Q   K   A   R   A
cag acc cac agt gaa gtg ata aaa gca gag agc aga aaa act gag ggc cag aaa ggg tga
```

Q T H S E V I K A E S R K T E G Q K G -
ctc ccc tga gtg tca tgc cac aag ttg eag tgc tgg agc cag gac acc ttc caa acc ggc
L P - **V** S C H K L X C W S Q D T F Q T G
tgt gcc tgc ctg caa gcc cca tcc tcc tcc atc ccc ctg agc agc cag act gca gag gga
C A C L Q A P S S S I P L S S Q T A E G
atg agc gga ccg aaa aca gtg tgg ccg caa ctt ccc cac tgc ttt gcg gga ggt gga ggg
M S G P K T V W P Q L P H C F A G G G G
agt ttc tag gct ctt tcc ctg ttg ggg aat taa gta gct ggg gat ccc act gag tct cca
S F - **A** L S L L G N - **V** A G D P T E S P
cca ccc tct cac cca ttt tcc acc cct ccc cca cca cat ata cat tct gct ccc cca gtg
P P S H P F S T P P P P H I H S A P P V
cca aat aac ttg cat ttt ctg gaa cac acc tgt ctg ctt acg gtc tct gtg cct ttc ttt
P N N L H F L E H T C L L T V S V P F F
gcc cac atc tcc cct ctt ctt gaa aca ccc ctc ctc ccc tcc agc gtg ggc tca tgt gac
A H I S P L L E T P L L P S S V G S C D
cta gag aag cac gtg gga gcc ctg tca tct ggg gga ctt ggg ttc aaa tcc cac ttc tgg
L E K H V G A L S S G G L G F K S H F W
tcc tta agt ctg tga ctc tgg gcc ttt gtt taa taa ctg gag gag ttt cat tag gaa aat
S L S L - **L** W A F V - - **L** E E F H - **E** N
ggc agt gac cac ata tgt ccc tgg agt gac tga tgt gcc acc cac aag ctt cta ttt ggt
G S D H I C P W S D - **C** A T H K L L F G
cag tgc tgg gtc cca tcg tcc cat cta gca tgc tcc tct tcc atc ctc gtg ccc cca ctc
Q C W V P S S H L A C S S S I L V P P L
agt gcc att gtc tct gcc tct cac cct ctg tgc tgc tat tcc tgc cct cat cac ctc ctc
S A I V S A S H P L C C Y S C P H H L L
cct agg gca gag gcc ccg tct tct tta cca tgc ccc agt gcc agc aca gtg ctt tgg caa
P R A E A P S S L P C P S A S T V L W Q
gtt att cag cac tgg ggg agt aga gtg tat gtg tgg tgg ggg agg ggg tgg aga atg ggt
V I Q H W G S R V Y V W W G R G W R **M** G
aag agg gtg atg gag act cag ggg gac ccc cag cta ctt aat acc cct gag gaa gtg tcg
K R V **M** E T Q G D P Q L L N T P E E V S
gcc gaa gtg gac cca ctc ctg tga cag ttc aca gcc tag aag agg cgt ctg taa cca cgg
A E V D P L L - **Q** F T A - **K** R R L - **P** R
tga cca gta agt gcc agc cct ggg gcc acg tgg ctg ttc tag aga agg aag aca gca cta
- **P** V S A S P G A T W L F - **R** R K T A L
ccc tga gag cca tgc tgg tgg ctt cat cag gaa ggg agt cag cag ggg gag gac tgg ggg
P - **E** P C W W L H Q E G S Q Q G E D W G
acc aag cgg tcc cta aga agc aga att tgc aat acc caa ggt ttt aga aag agc aac ctg
T K R S L R S R I C N T Q G F R K S N L
gca ggg cca ctg cac aat gag atc aca gac tca cct ccc agc tgt gag tgc agc tca ccc
A G P L H N E I T D S P P S C E C S S P
cac ctg ctc tga ccc agg cac cgg tta tga aga aga caa gca gct cta cag agg ggc ctc
H L L - **P** R H R L - **R** R Q A A L Q R G L
aag aaa tcc ttc gaa aca gaa atc aaa gtc ttg ctg gaa ggg ttc tgg gca ccc tgg act
K K S F E T E I K V L L E G F W A P W T
gtc cac atg gga ggc tgt tgg tgc gtg tgc cta cgt gtg tcc atc agt gga tgc acg aag
V H **M** G G C W C V C L R V S I S G C T K
aag gaa atg aat tga ttc cat tct tga tgt gac ttt cag ctg tec agc tgt ctg cac tcc
K E **M** N - **F** H S - **C** D F Q L X S C L H S
cag acg ggg tga tgg cct ggc acc aac gga gca tta ccc tga tga atc atg gtc gag gta
Q T G - **W** P G T N G A L P - - **I** **M** V E V
ctt ttc cac tgg gcc agc cgc cac agc ccc ttc tcc atc ctt cgg aga gct ctg tca cct
L F H W A S R H S P F S I L R R A L S P
cgc ctt gta gca ctt gtg tct ctc cca ctg gcc tcc act tct tgt ggg cac ttt ctg gat
R L V A L V S L P L A S T S C G H F L D
gcc cag gcc taa tcc agc gct ggc act cag tta att ctc tct tcc ttt aaa ggg aac aat
A Q A - **S** S A G T Q L I L S S F K G N N
tac cac ctt gag gaa gga ggc ctg aaa gct aag tct ttg cta ttg cat ccg cct tct tct
Y H L E E G G L K A K S L L L H P P S S
cac gaa gac ctg cag ctt gaa agg agg gga ggg aga aat agc cct aag ctc cag gaa gac
H E D L Q L E R R G G R N S P K L Q E D
caa gcc cag cct ctg tga tga ttc cga gga aat tca gga aat cct tgc taa tgg cca aac
Q A Q P L - - **F** R G N S G N P C - **W** P N
ttc ccg ggt ttg aga tgc aaa agg aaa tca gat cgc agg gta atg ctt tta cac taa gca
F P G L R C K R K S D R R V **M** L L H - **A**
aga aat tgc agg ata taa agc tgt gca gac cat tgc ctc tga tta aga aag cga ttc tga
R N C R I - **S** C A D H C L - **L** R K R F -
gat tcc agc ctg gaa gga acc ggg ccc cag tgt taa ctg tgg tta att ctg ggt gga ggg
D S S L E G T G P Q C - **L** W L I L G G G
gtg atg ggt gat aat ttc tcc ttt ttc ttt tct gta cta acc att aat act ggg ggg atg
V **M** G D N F S F F F S V L T I N T G G **M**
ggc agt taa aaa tca aag gaa gaa aac ata aag aaa gcc aaa tcc ttt tcc atc tgt agc
G S - **K** S K E E N I K K A K S F S I C S
cat tca agc tac tgc ctg gac acg ctc tcc ttg aag ctg ggg ctc ctc tct cag ccc cac
H S S Y C L D T L S L K L G L L S Q P H
cta aca tgg ccc acc tcc ttc ctg ggg tca ctc cgt gtt cct caa gca ctt caa act cat
L T W P T S F L G S L R V P Q A L Q T H
cct ctc ctg cct ctg agc tga gca tat ggg cct ctc ccc ttc ctt taa aag ctc cat ccg
P L L P L S - **A** Y G P L P F L - **K** L H P
tga ctg cct tct ggg ggt cag cca cat agc tga gtg agg acc aca tgc ttc aca cca aat
- **L** P S G G Q P H S - **V** R T T C F T P N

gat aca aga agc ttg ttc caa atc tat gtg ggt ttt ttg aca ttt tag aga gca gat gct
D T R S L F Q I Y V G F L T F - R A D A
ttc cca tgc ttt cta att ccc tat tac atc tat aca tct ctt tta aaa gat cct gca agt
F P C F L I P Y Y I Y T S L L K D P A S
cct ttt agt gtc tgg aca cag gtt att ccc tcc ctt tct ttt ttc cca att tca cct gcc
P F S V W T Q V I P S L S F F P I S P A
cca gtc ctc ctg gtc agt gct tct gtt tcc tgc aca agg aaa cag cct tgc aaa cat ggt
P V L V S A S V S C T R K Q P C K H G
gaa ccc tga aag cat gct gga aat gaa aga agc cag gta caa aag aca aca tac tat atg
E P - K H A G N E R S Q V Q K T T Y Y M
att tca ttc aca aga aat gtg tgt aac aga aaa att tac aga gac aga aaa taa att cct
I S F T R N V C N R K I Y R D R K - I P
agt tgc tta ggg ctg gga gag tgg ggt gag gcc tct cta cag aga tgg caa tgc cca gga
S C L G L G E W G E A S L Q R W Q C P G
ttc aat ctg aag atg ttg ttt aga tat cac agc ata gtc gga caa tag tga gga cag tct
F N L K M L F R Y H S I V G Q - - G Q S
ggc att ggc gta cag atg aac aaa tag att tgt gca ata gaa gaa gcc aaa cca gat tcc
G I G V Q M N K - I C A I E E A K P D S
agc cta cct cca gtc acc tga ttt aac aaa gtc aaa gtc aca aat caa tag gga gag gat
S L P P V T - F N K V K V T N Q - G E D
ggt ctt ttc agt aaa tga tac cgt atc aat tga ctc gtc ata tac aga aca aaa tga act
G L F S K - Y R I N - L V I Y R T K - T
ttg gtc cca tac aca cac aag tca taa aag ttc act caa agt gaa tga tag acc tac att
L V P Y T H K S - K F T Q S E - - T Y I
tga aaa caa aag caa tgc agg atc cag gag aaa ggg tcc agg cag gag aat att ttt atg
- K Q K Q C R I Q E K G S R Q E N I F M
acc ttg gac taa gca aga gat tca taa aca gga cac aaa acc act aac cac caa aaa aaa
T L D - A R D S - T G H K T T N H Q K K
gga ctg ata aac atg aca att aaa atg aag aat tta tgt tta tca aaa aca aaa agg gga
G L I N M T I K M K N L C L S K T K R G
acc atc gac tgg gag aac ata gtt aca aca ctt gta tcc aac tga gaa ttt ata ttc agg
T I D W E N I V T T L V S N - E F I F R
tta gaa gac tca aaa aca aaa agg caa gca atc cac taa gaa caa att ctg ggc aga aga
L E D S K T K R Q A I H - E Q I L G R R
ctt cac aaa aga tct gca aat gac caa tga cat cag gta aac atg gat tga aac aac aag
L H K R S A N D Q - H Q V N M D - N N K
aag aca cac cca caa gat ccg tga caa ttt tga aaa tga aac cct gag gca ggc aga att
K T H P Q D P - Q F - K - N P E A G R I
cta agg tca tgt ccc aga ttc ccc ctc ttg gtg tcc gag ccc tgc aca gtc cac ttg agt
L R S C P R F P L L V S E P C T V H L S
agg ggc cag acc tgt gga tat gat gga tgt ctc ttc tgt gat cag gtt aca tca tat gcc
R G Q T C G Y D G C L F C D Q V T S Y A
aaa gat gga ggg att ttg tga tta agt ctc taa tca gtt tac ctt caa gtt cat caa aag
K D G G I L - L S L - S V Y L Q V H Q K
cga gag tgg cct agg ttg gcc tgg ctt act cag gtg agg cct tta aaa gag agc tca agc
R E W P R L A W L T Q V R P L K E S S S
ctg ccc tga gct tgg aga gat ttt cct gct agc ctt gaa gaa aca aac agc tgt gag ttc
L P - A W R D F P A S L E E T N S C E F
tgc agc ttc aca gaa atg aat ttg gcc gac aat cat gtg agc tcc att aga aat gca ccc
C S F T E M N L A D N H V S S I R N A P
taa gct ctc tca acc tct ccc atc ccc cac agc acc tat tta ctg att atc tgt aca tga
- A L S T S P I P H S T Y L L I I C T -
tag cag ggc agg aac tag act ctc agc ccc ctg agg aca gac ctt gtc tgt cag tgc att
- Q G R N - T L S P L R T D L V C Q C I
tca ctg tct gtc cca gca cct tgc aca gag cct ggc aca tat caa gaa cac ggc aca ttt
S L S V P A P C T E P G T Y Q E H G T F
tta ttg aga cag act cct caa agc tgg aat cta aaa cca atg gca cct aat ctc agc caa
L L R Q T P Q S W N L K P M A P N L S Q
aaa ggt taa tgt gac acc tgt gct ttt att acc cat ttc tgc aga tgg aag ggt tgg ttg acc>atc, Thr>Ile
K G - C D T C A F I T H F C R W K G W L
ttt gtg gct atc tca agt gaa atg cag aat tag tga ata aaa ttt tca tct gaa ttt aaa
F V A I S S E M Q N - - I K F S S E F K
gaa aat ctt aa
E N L

5'3' Frame 2

gggc gcg ccc ggg cac ggc ggt gct ggc ggg gcg cgg gcg ggg cag cgc aca ggc cgg ccc
G A P G H G G A G G A R A G Q R T G R P
tct cgc ggg act cag ttt ccc cgt ctg cgc gcc cca cat att cgc ttt ccg cag gtt ctc
S R G T Q F P R L R A P H I R F P Q V L
tcg ctg aga ttc gcg tcc ttc aaa cta atg gaa ctt tgc aag ggg gcc ctg cgg cgg ctt
S L R F A S F K L M E L C K G A L R R L
cgc gtg gga gcg ccg ggg aaa gtt cct gca aat cgc cgg act ggg ggc ccg ccc ggg aag
R V G A P G K V P A N R R T G G P P G K
ctc gga ctg ggc cgg gca ggg act gca ggg gcc tct gcg cag acc cgg ggc gtt gcg gca
L G L G R A G T A G A S A Q T R G V A A
ccg cgg acc ccg gtc acg cgc tgg ttc tgc ccc cgg tac ccc gca cgc ggg acc ctg acc
P R T P V T R W F C P R Y P A R G T L T
gcg ggg cct ctg cgg ccg gac gaa ggc agc gtc cgc gga gct ggc aga aag ccc gcg ccc
A G P L R P D E G S V R G A G R K P A P
aga ccc aca gtg aag tga taa aag cag aga gca gaa aaa ctg agg gcc aga aag ggt gac

```
 R   P   T   V   K   -   -   K   Q   R   A   E   K   L   R   A   R   K   G   D
tcc cct gag tgt cat gcc aca agt tge agt gct gga gcc agg aca cct tcc aaa ccg gct
 S   P   E   C   H   A   T   S   X   S   A   G   A   R   T   P   S   K   P   A
gtg cct gcc tgc aag ccc cat cct cct cca tcc ccc tga gca gcc aga ctg cag agg gaa
 V   P   A   C   K   P   H   P   P   P   S   P   -   A   A   R   L   Q   R   E
tga gcg gac cga aaa cag tgt ggc cgc aac ttc ccc act gct ttg cgg gag gtg gag gga
 -   A   D   R   K   Q   C   G   R   N   F   P   T   A   L   R   E   V   E   G
gtt tct agg ctc ttt ccc tgt tgg gga att aag tag ctg ggg atc cca ctg agt ctc cac
 V   S   R   L   F   P   C   W   G   I   K   -   L   G   I   P   L   S   L   H
cac cct ctc acc cat ttt cca ccc ctc ccc cac cac ata tac att ctg ctc ccc cag tgc
 H   P   L   T   H   F   P   P   L   P   H   H   I   Y   I   L   L   P   Q   C
caa ata act tgc att ttc tgg aac aca cct gtc tgc tta cgg tct ctg tgc ctt tct ttg
 Q   I   T   C   I   F   W   N   T   P   V   C   L   R   S   L   C   L   S   L
ccc aca tct ccc ctc ttc ttg aaa cac ccc tcc tcc cct cca gcg tgg gct cat gtg acc
 P   T   S   P   L   F   L   K   H   P   S   S   P   P   A   W   A   H   V   T
tag aga agc acg tgg gag ccc tgt cat ctg ggg gac ttg ggt tca aat ccc act tct ggt
 -   R   S   T   W   E   P   C   H   L   G   D   L   G   S   N   P   T   S   G
cct taa gtc tgt gac tct ggg cct ttg ttt aat aac tgg agg agt ttc att agg aaa atg
 P   -   V   C   D   S   G   P   L   F   N   N   W   R   S   F   I   R   K   M
gca gtg acc aca tat gtc cct gga gtg act gat gtg cca ccc aca agc ttc tat ttg gtc
 A   V   T   T   Y   V   P   G   V   T   D   V   P   P   T   S   F   Y   L   V
agt gct ggg tcc cat cgt ccc atc tag cat gct cct ctt cca tcc tcg tgc ccc cac tca
 S   A   G   S   H   R   P   I   -   H   A   P   L   P   S   S   C   P   H   S
gtg cca ttg tct ctg cct ctc acc ctc tgt gct gct att cct gcc ctc atc acc tcc tcc
 V   P   L   S   L   P   L   T   L   C   A   A   I   P   A   L   I   T   S   S
cta ggg cag agg ccc cgt ctt ctt tac cat gcc cca gtg cca gca cag tgc ttt ggc aag
 L   G   Q   R   P   R   L   L   Y   H   A   P   V   P   A   Q   C   F   G   K
tta ttc agc act ggg gga gta gag tgt atg tgt ggt ggg gga ggg ggt gga gaa tgg gta
 L   F   S   T   G   G   V   E   C   M   C   G   G   G   G   G   G   E   W   V
aga ggg tga tgg aga ctc agg ggg acc ccc agc tac tta ata ccc ctg agg aag tgt cgg
 R   G   -   W   R   L   R   G   T   P   S   Y   L   I   P   L   R   K   C   R
ccg aag tgg acc cac tcc tgt gac agt tca cag cct aga aga ggc gtc tgt aac cac ggt
 P   K   W   T   H   S   C   D   S   S   Q   P   R   R   G   V   C   N   H   G
gac cag taa gtg cca gcc ctg ggg cca cgt ggc tgt tct aga gaa gga aga cag cac tac
 D   Q   -   V   P   A   L   G   P   R   G   C   S   R   E   G   R   Q   H   Y
cct gag agc cat gct ggt ggc ttc atc agg aag gga gtc agc agg ggg agg act ggg gga
 P   E   S   H   A   G   G   F   I   R   K   G   V   S   R   G   R   T   G   G
cca agc ggt ccc taa gaa gca gaa ttt gca ata ccc aag gtt tta gaa aga gca acc tgg
 P   S   G   P   -   E   A   E   F   A   I   P   K   V   L   E   R   A   T   W
cag ggc cac tgc aca atg aga tca cag act cac ctc cca gct gtg agt gca gct cac ccc
 Q   G   H   C   T   M   R   S   Q   T   H   L   P   A   V   S   A   A   H   P
acc tgc tct gac cca ggc acc ggt tat gaa gaa gac aag cag ctc tac aga ggg gcc tca
 T   C   S   D   P   G   T   G   Y   E   E   D   K   Q   L   Y   R   G   A   S
aga aat cct tcg aaa cag aaa tca aag tct tgc tgg aag ggt tct ggg cac cct gga ctg
 R   N   P   S   K   Q   K   S   K   S   C   W   K   G   S   G   H   P   G   L
tcc aca tgg gag gct gtt ggt gcg tgt gcc tac gtg tgt cca tca gtg gat gca cga aga
 S   T   W   E   A   V   G   A   C   A   Y   V   C   P   S   V   D   A   R   R
agg aaa tga att gat tcc att ctt gat gtg act ttc agc tgt eca gct gtc tgc act ccc
 R   K   -   I   D   S   I   L   D   V   T   F   S   C   X   A   V   C   T   P
aga cgg ggt gat ggc ctg gca cca acg gag cat tac cct gat gaa tca tgg tcg agg tac
 R   R   G   D   G   L   A   P   T   E   H   Y   P   D   E   S   W   S   R   Y
ttt tcc act ggg cca gcc gcc aca gcc cct tct cca tcc ttc gga gag ctc tgt cac ctc
 F   S   T   G   P   A   A   T   A   P   S   P   S   F   G   E   L   C   H   L
gcc ttg tag cac ttg tgt ctc tcc cac tgg cct cca ctt ctt gtg ggc act ttc tgg atg
 A   L   -   H   L   C   L   S   H   W   P   P   L   L   V   G   T   F   W   M
ccc agg cct aat cca gcg ctg gca ctc agt taa ttc tct ctt cct tta aag gga aca att
 P   R   P   N   P   A   L   A   L   S   -   F   S   L   P   L   K   G   T   I
acc acc ttg agg aag gag gcc tga aag cta agt ctt tgc tat tgc atc cgc ctt ctt ctc
 T   T   L   R   K   E   A   -   K   L   S   L   C   Y   C   I   R   L   L   L
acg aag acc tgc agc ttg aaa gga ggg gag gga gaa ata gcc cta agc tcc agg aag acc
 T   K   T   C   S   L   K   G   G   E   G   E   I   A   L   S   S   R   K   T
aag ccc agc ctc tgt gat gat tcc gag gaa att cag gaa atc ctt gct aat ggc caa act
 K   P   S   L   C   D   D   S   E   E   I   Q   E   I   L   A   N   G   Q   T
tcc cgg gtt tga gat gca aaa gga aat cag atc gca ggg taa tgc ttt tac act aag caa
 S   R   V   -   D   A   K   G   N   Q   I   A   G   -   C   F   Y   T   K   Q
gaa att gca gga tat aaa gct gtg cag acc att gcc tct gat taa gaa agc gat tct gag
 E   I   A   G   Y   K   A   V   Q   T   I   A   S   D   -   E   S   D   S   E
att cca gcc tgg aag gaa ccg ggc ccc agt gtt aac tgt ggt taa ttc tgg gtg gag ggg
 I   P   A   W   K   E   P   G   P   S   V   N   C   G   -   F   W   V   E   G
tga tgg gtg ata att tct cct ttt tct ttt ctg tac taa cca tta ata ctg ggg gga tgg
 -   W   V   I   I   S   P   F   S   F   L   Y   -   P   L   I   L   G   G   W
gca gtt aaa aat caa agg aag aaa aca taa aga aag cca aat cct ttt cca tct gta gcc
 A   V   K   N   Q   R   K   K   T   -   R   K   P   N   P   F   P   S   V   A
att caa gct act gcc tgg aca cgc tct cct tga agc tgg ggc tcc tct ctc agc ccc acc
 I   Q   A   T   A   W   T   R   S   P   -   S   W   G   S   S   L   S   P   T
taa cat ggc cca cct cct tcc tgg ggt cac tcc gtg ttc ctc aag cac ttc aaa ctc atc
 -   H   G   P   P   P   S   W   G   H   S   V   F   L   K   H   F   K   L   I
ctc tcc tgc ctc tga gct gag cat atg ggc ctc tcc cct tcc ttt aaa agc tcc atc cgt
 L   S   C   L   -   A   E   H   M   G   L   S   P   S   F   K   S   S   I   R
gac tgc ctt ctg ggg gtc agc cac ata gct gag tga gga cca cat gct tca cac caa atg
 D   C   L   L   G   V   S   H   I   A   E   -   G   P   H   A   S   H   Q   M
```

ata caa gaa gct tgt tcc aaa tct atg tgg gtt ttt tga cat ttt aga gag cag atg ctt
I Q E A C S K S M W V F - H F R E Q M L
tcc cat gct ttc taa ttc cct att aca tct ata cat ctc ttt taa aag atc ctg caa gtc
S H A F - F P I T S I H L F - K I L Q V
ctt tta gtg tct gga cac agg tta ttc cct ccc ttt ctt ttt tcc caa ttt cac ctg ccc
L L V S G H R L F P P F L F S Q F H L P
cag tcc tcc tgg tca gtg ctt ctg ttt cct gca caa gga aac agc ctt gca aac atg gtg
Q S S W S V L L F P A Q G N S L A N M V
aac cct gaa agc atg ctg gaa atg aaa gaa gcc agg tac aaa aga caa cat act ata tga
N P E S M L E M K E A R Y K R Q H T I -
ttt cat tca caa gaa atg tgt gta aca gaa aaa ttt aca gag aca gaa aat aaa ttc cta
F H S Q E M C V T E K F T E T E N K F L
gtt gct tag ggc tgg gag agt ggg gtg agg cct ctc tac aga gat ggc aat gcc cag gat
V A - G W E S G V R P L Y R D G N A Q D
tca atc tga aga tgt tgt tta gat atc aca gca tag tcg gac aat agt gag gac agt ctg
S I - R C C L D I T A - S D N S E D S L
gca ttg gcg tac aga tga aca aat aga ttt gtg caa tag aag aag cca aac cag att cca
A L A Y R - T N R F V Q - K K P N Q I P
gcc tac ctc cag tca cct gat tta aca aag tca aag tca caa atc aat agg gag agg atg
A Y L Q S P D L T K S K S Q I N R E R M
gtc ttt tca gta aat gat acc gta tca att gac tcg tca tat aca gaa caa aat gaa ctt
V F S V N D T V S I D S S Y T E Q N E L
tgg tcc cat aca cac aca agt cat aaa agt tca ctc aaa gtg aat gat aga cct aca ttt
W S H T H T S H K S S L K V N D R P T F
gaa aac aaa agc aat gca gga tcc agg aga aag ggt cca ggc agg aga ata ttt tta tga
E N K S N A G S R R K G P G R R I F L -
cct tgg act aag caa gag att cat aaa cag gac aca aaa cca cta acc acc aaa aaa aag
P W T K Q E I H K Q D T K P L T T K K K
gac tga taa aca tga caa tta aaa tga aga att tat gtt tat caa aaa caa aaa ggg gaa
D - - T - Q L K - R I Y V Y Q K Q K G E
cca tcg act ggg aga aca tag tta caa cac ttg tat cca act gag aat tta tat tca ggt
P S T G R T - L Q H L Y P T E N L Y S G
tag aag act caa aaa caa aaa ggc aag caa tcc act aag aac aaa ttc tgg gca gaa gac
- K T Q K Q K G K Q S T K N K F W A E D
ttc aca aaa gat ctg caa atg acc aat gac atc agg taa aca tgg att gaa aca aca aga
F T K D L Q M T N D I R - T W I E T T R
aga cac acc cac aag atc cgt gac aat ttt gaa aat gaa acc ctg agg cag gca gaa ttc
R H T H K I R D N F E N E T L R Q A E F
taa ggt cat gtc cca gat tcc ccc tct tgg tgt ccg agc cct gca cag tcc act tga gta
- G H V P D S P S W C P S P A Q S T - V
ggg gcc aga cct gtg gat atg atg gat gtc tct tct gtg atc agg tta cat cat atg cca
G A R P V D M M D V S S V I R L H H M P
aag atg gag gga ttt tgt gat taa gtc tct aat cag ttt acc ttc aag ttc atc aaa agc
K M E G F C D - V S N Q F T F K F I K S
gag agt ggc cta ggt tgg cct ggc tta ctc agg tga ggc ctt taa aag aga gct caa gcc
E S G L G W P G L L R - G L - K R A Q A
tgc cct gag ctt gga gag att ttc ctg cta gcc ttg aag aaa caa aca gct gtg agt tct
C P E L G E I F L L A L K K Q T A V S S
gca gct tca cag aaa tga att tgg ccg aca atc atg tga gct cca tta gaa atg cac cct
A A S Q K - I W P T I M - A P L E M H P
aag ctc tct caa cct ctc cca tcc ccc aca gca cct att tac tga tta tct gta cat gat
K L S Q P L P S P T A P I Y - L S V H D
agc agg gca gga act aga ctc tca gcc ccc tga gga cag acc ttg tct gtc agt gca ttt
S R A G T R L S A P - G Q T L S V S A F
cac tgt ctg tcc cag cac ctt gca cag agc ctg gca cat atc aag aac acg gca cat ttt
H C L S Q H L A Q S L A H I K N T A H F
tat tga gac aga ctc ctc aaa gct gga atc taa aac caa tgg cac cta atc tca gcc aaa
Y - D R L L K A G I - N Q W H L I S A K
aag gtt aat gtg aca cct gtg ctt tta tta ccc att tct gca gat gga agg gtt ggt tgt
K V N V T P V L L L P I S A D G R V G C
ttg tgg cta tct caa gtg aaa tgc aga att agt gaa taa aat ttt cat ctg aat tta aag
L W L S Q V K C R I S E - N F H L N L K
aaa atc tta a
K I L

5'3' Frame 3

gggcg cgc ccg ggc acg gcg gtg ctg gcg ggg cgc ggg cgg ggc agc gca cag gcc ggc cct
A R P G T A V L A G R G R G S A Q A G P
ctc gcg gga ctc agt ttc ccc gtc tgc gcg ccc cac ata ttc gct ttc cgc agg ttc tct
L A G L S F P V C A P H I F A F R R F S
cgc tga gat tcg cgt cct tca aac taa tgg aac ttt gca agg ggg ccc tgc ggc ggc ttc
R - D S R P S N - W N F A R G P C G G F
gcg tgg gag cgc cgg gga aag ttc ctg caa atc gcc gga ctg ggg gcc cgc ccg gga agc
A W E R R G K F L Q I A G L G A R P G S
tcg gac tgg gcc ggg cag gga ctg cag ggg cct ctg cgc aga ccc ggg gcg ttg cgg cac
S D W A G Q G L Q G P L R R P G A L R H
cgc gga ccc cgg tca cgc gct ggt tct gcc ccc ggt acc ccg cac gcg gga ccc tga ccg
R G P R S R A G S A P G T P H A G P - P
cgg ggc ctc tgc ggc cgg acg aag gca gcg tcc gcg gag ctg gca gaa agc ccg cgc cca
R G L C G R T K A A S A E L A E S P R P
gac cca cag tga agt gat aaa agc aga gag cag aaa aac tga ggg cca gaa agg gtg act

```
 D   P   Q   -   S   D   K   S   R   E   Q   K   N   -   G   P   E   R   V   T
ccc ctg agt gtc atg cca caa gtt gea gtg ctg gag cca gga cac ctt cca aac cgg ctg
 P   L   S   V   M   P   Q   V   X   V   L   E   P   G   H   L   P   N   R   L
tgc ctg cct gca agc ccc atc ctc ctc cat ccc cct gag cag cca gac tgc aga ggg aat
 C   L   P   A   S   P   I   L   L   H   P   P   E   Q   P   D   C   R   G   N
gag cgg acc gaa aac agt gtg gcc gca act tcc cca ctg ctt tgc ggg agg tgg agg gag
 E   R   T   E   N   S   V   A   A   T   S   P   L   L   C   G   R   W   R   E
ttt cta ggc tct ttc cct gtt ggg gaa tta agt agc tgg gga tcc cac tga gtc tcc acc
 F   L   G   S   F   P   V   G   E   L   S   S   W   G   S   H   -   V   S   T
acc ctc tca ccc att ttc cac ccc tcc ccc acc aca tat aca ttc tgc tcc ccc agt gcc
 T   L   S   P   I   F   H   P   S   P   T   T   Y   T   F   C   S   P   S   A
aaa taa ctt gca ttt tct gga aca cac ctg tct gct tac ggt ctc tgt gcc ttt ctt tgc
 K   -   L   A   F   S   G   T   H   L   S   A   Y   G   L   C   A   F   L   C
cca cat ctc ccc tct tct tga aac acc cct cct ccc ctc cag cgt ggg ctc atg tga cct
 P   H   L   P   S   S   -   N   T   P   P   P   L   Q   R   G   L   M   -   P
aga gaa gca cgt ggg agc cct gtc atc tgg ggg act tgg gtt caa atc cca ctt ctg gtc
 R   E   A   R   G   S   P   V   I   W   G   T   W   V   Q   I   P   L   L   V
ctt aag tct gtg act ctg ggc ctt tgt tta ata act gga gga gtt tca tta gga aaa tgg
 L   K   S   V   T   L   G   L   C   L   I   T   G   G   V   S   L   G   K   W
cag tga cca cat atg tcc ctg gag tga ctg atg tgc cac cca caa gct tct att tgg tca
 Q   -   P   H   M   S   L   E   -   L   M   C   H   P   Q   A   S   I   W   S
gtg ctg ggt ccc atc gtc cca tct agc atg ctc ctc ttc cat cct cgt gcc ccc act cag
 V   L   G   P   I   V   P   S   S   M   L   L   F   H   P   R   A   P   T   Q
tgc cat tgt ctc tgc ctc tca ccc tct gtg ctg cta ttc ctg ccc tca tca cct cct ccc
 C   H   C   L   C   L   S   P   S   V   L   L   F   L   P   S   S   P   P   P
tag ggc aga ggc ccc gtc ttc ttt acc atg ccc cag tgc cag cac agt gct ttg gca agt
 -   G   R   G   P   V   F   F   T   M   P   Q   C   Q   H   S   A   L   A   S
tat tca gca ctg ggg gag tag agt gta tgt gtg gtg ggg gag ggg gtg gag aat ggg taa
 Y   S   A   L   G   E   -   S   V   C   V   V   G   E   G   V   E   N   G   -
gag ggt gat gga gac tca ggg gga ccc cca gct act taa tac ccc tga gga agt gtc ggc
 E   G   D   G   D   S   G   G   P   P   A   T   -   Y   P   -   G   S   V   G
cga agt gga ccc act cct gtg aca gtt cac agc cta gaa gag gcg tct gta acc acg gtg
 R   S   G   P   T   P   V   T   V   H   S   L   E   E   A   S   V   T   T   V
acc agt aag tgc cag ccc tgg ggc cac gtg gct gtt cta gag aag gaa gac agc act acc
 T   S   K   C   Q   P   W   G   H   V   A   V   L   E   K   E   D   S   T   T
ctg aga gcc atg ctg gtg gct tca tca gga agg gag tca gca ggg gga gga ctg ggg gac
 L   R   A   M   L   V   A   S   S   G   R   E   S   A   G   G   G   L   G   D
caa gcg gtc cct aag aag cag aat ttg caa tac cca agg ttt tag aaa gag caa cct ggc
 Q   A   V   P   K   K   Q   N   L   Q   Y   P   R   F   -   K   E   Q   P   G
agg gcc act gca caa tga gat cac aga ctc acc tcc cag ctg tga gtg cag ctc acc cca
 R   A   T   A   Q   -   D   H   R   L   T   S   Q   L   -   V   Q   L   T   P
cct gct ctg acc cag gca ccg gtt atg aag aag aca agc agc tct aca gag ggg cct caa
 P   A   L   T   Q   A   P   V   M   K   K   T   S   S   S   T   E   G   P   Q
gaa atc ctt cga aac aga aat caa agt ctt gct gga agg gtt ctg ggc acc ctg gac tgt
 E   I   L   R   N   R   N   Q   S   L   A   G   R   V   L   G   T   L   D   C
cca cat ggg agg ctg ttg gtg cgt gtg cct acg tgt gtc cat cag tgg atg cac gaa gaa
 P   H   G   R   L   L   V   R   V   P   T   C   V   H   Q   W   M   H   E   E
gga aat gaa ttg att cca ttc ttg atg tga ctt tca gct gte cag ctg tct gca ctc cca
 G   N   E   L   I   P   F   L   M   -   L   S   A   X   Q   L   S   A   L   P
gac ggg gtg atg gcc tgg cac caa cgg agc att acc ctg atg aat cat ggt cga ggt act   SNP G/A=C/T, Thr>Ile
 D   G   V   M   A   W   H   Q   R   S   I   T   L   M   N   H   G   R   G   T
ttt cca ctg ggc cag ccg cca cag ccc ctt ctc cat cct tcg gag agc tct gtc acc tcg
 F   P   L   G   Q   P   P   Q   P   L   L   H   P   S   E   S   S   V   T   S
cct tgt agc act tgt gtc tct ccc act ggc ctc cac ttc ttg tgg gca ctt tct gga tgc
 P   C   S   T   C   V   S   P   T   G   L   H   F   L   W   A   L   S   G   C
cca ggc cta atc cag cgc tgg cac tca gtt aat tct ctc ttc ctt taa agg gaa caa tta
 P   G   L   I   Q   R   W   H   S   V   N   S   L   F   L   -   R   E   Q   L
cca cct tga gga agg agg cct gaa agc taa gtc ttt gct att gca tcc gcc ttc ttc tca
 P   P   -   G   R   R   P   E   S   -   V   F   A   I   A   S   A   F   F   S
cga aga cct gca gct tga aag gag ggg agg gag aaa tag ccc taa gct cca gga aga cca
 R   R   P   A   A   -   K   E   G   R   E   K   -   P   -   A   P   G   R   P
agc cca gcc tct gtg atg att ccg agg aaa ttc agg aaa tcc ttg cta atg gcc aaa ctt
 S   P   A   S   V   M   I   P   R   K   F   R   K   S   L   L   M   A   K   L
ccc ggg ttt gag atg caa aag gaa atc aga tcg cag ggt aat gct ttt aca cta agc aag
 P   G   F   E   M   Q   K   E   I   R   S   Q   G   N   A   F   T   L   S   K
aaa ttg cag gat ata aag ctg tgc aga cca ttg cct ctg att aag aaa gcg att ctg aga
 K   L   Q   D   I   K   L   C   R   P   L   P   L   I   K   K   A   I   L   R
ttc cag cct gga agg aac cgg gcc cca gtg tta act gtg gtt aat tct ggg tgg agg ggt
 F   Q   P   G   R   N   R   A   P   V   L   T   V   V   N   S   G   W   R   G
gat ggg tga taa ttt ctc ctt ttt ctt ttc tgt act aac cat taa tac tgg ggg gat ggg
 D   G   -   -   F   L   L   F   L   F   C   T   N   H   -   Y   W   G   D   G
cag tta aaa atc aaa gga aga aaa cat aaa gaa agc caa atc ctt ttc cat ctg tag cca
 Q   L   K   I   K   G   R   K   H   K   E   S   Q   I   L   F   H   L   -   P
ttc aag cta ctg cct gga cac gct ctc ctt gaa gct ggg gct cct ctc tca gcc cca cct
 F   K   L   L   P   G   H   A   L   L   E   A   G   A   P   L   S   A   P   P
aac atg gcc cac ctc ctt cct ggg gtc act ccg tgt tcc tca agc act tca aac tca tcc
 N   M   A   H   L   L   P   G   V   T   P   C   S   S   S   T   S   N   S   S
tct cct gcc tct gag ctg agc ata tgg gcc tct ccc ctt cct tta aaa gct cca tcc gtg
 S   P   A   S   E   L   S   I   W   A   S   P   L   P   L   K   A   P   S   V
act gcc ttc tgg ggg tca gcc aca tag ctg agt gag gac cac atg ctt cac acc aaa tga
 T   A   F   W   G   S   A   T   -   L   S   E   D   H   M   L   H   T   K   -
```

tac aag aag ctt gtt cca aat cta tgt ggg ttt ttt gac att tta gag agc aga tgc ttt
Y K K L V P N L C G F F D I L E S R C F
ccc atg ctt tct aat tcc cta tta cat cta tac atc tct ttt aaa aga tcc tgc aag tcc
P M L S N S L L H L Y I S F K R S C K S
ttt tag tgt ctg gac aca ggt tat tcc ctc cct ttc ttt ttt ccc aat ttc acc tgc ccc
F - C L D T G Y S L P F F F P N F T C P
agt cct cct ggt cag tgc ttc tgt ttc ctg cac aag gaa aca gcc ttg caa aca tgg tga
S P P G Q C F C F L H K E T A L Q T W -
acc ctg aaa gca tgc tgg aaa tga aag aag cca ggt aca aaa gac aac ata cta tat gat
T L K A C W K - K K P G T K D N I L Y D
ttc att cac aag aaa tgt gtg taa cag aaa aat tta cag aga cag aaa ata aat tcc tag
F I H K K C V - Q K N L Q R Q K I N S -
ttg ctt agg gct ggg aga gtg ggg tga ggc ctc tct aca gag atg gca atg ccc agg att
L L R A G R V G - G L S T E M A M P R I
caa tct gaa gat gtt gtt tag ata tca cag cat agt cgg aca ata gtg agg aca gtc tgg
Q S E D V V - I S Q H S R T I V R T V W
cat tgg cgt aca gat gaa caa ata gat ttg tgc aat aga aga agc caa acc aga ttc cag
H W R T D E Q I D L C N R R S Q T R F Q
cct acc tcc agt cac ctg att taa caa agt caa agt cac aaa tca ata ggg aga gga tgg
P T S S H L I - Q S Q S H K S I G R G W
tct ttt cag taa atg ata ccg tat caa ttg act cgt cat ata cag aac aaa atg aac ttt
S F Q - M I P Y Q L T R H I Q N K M N F
ggt ccc ata cac aca caa gtc ata aaa gtt cac tca aag tga atg ata gac cta cat ttg
G P I H T Q V I K V H S K - M I D L H L
aaa aca aaa gca atg cag gat cca gga gaa agg gtc cag gca gga gaa tat ttt tat gac
K T K A M Q D P G E R V Q A G E Y F Y D
ctt gga cta agc aag aga ttc ata aac agg aca caa aac cac taa cca cca aaa aaa agg
L G L S K R F I N R T Q N H - P P K K R
act gat aaa cat gac aat taa aat gaa gaa ttt atg ttt atc aaa aac aaa aag ggg aac
T D K H D N - N E E F M F I K N K K G N
cat cga ctg gga gaa cat agt tac aac act tgt atc caa ctg aga att tat att cag gtt
H R L G E H S Y N T C I Q L R I Y I Q V
aga aga ctc aaa aac aaa aag gca agc aat cca cta aga aca aat tct ggg cag aag act
R R L K N K K A S N P L R T N S G Q K T
tca caa aag atc tgc aaa tga cca atg aca tca ggt aaa cat gga ttg aaa caa caa gaa
S Q K I C K - P M T S G K H G L K Q Q E
gac aca ccc aca aga tcc gtg aca att ttg aaa atg aaa ccc tga ggc agg cag aat tct
D T P T R S V T I L K M K P - G R Q N S
aag gtc atg tcc cag att ccc cct ctt ggt gtc cga gcc ctg cac agt cca ctt gag tag
K V M S Q I P P L G V R A L H S P L E -
ggg cca gac ctg tgg ata tga tgg atg tct ctt ctg tga tca ggt tac atc ata tgc caa
G P D L W I - W M S L L - S G Y I I C Q
aga tgg agg gat ttt gtg att aag tct cta atc agt tta cct tca agt tca tca aaa gcg
R W R D F V I K S L I S L P S S S S K A
aga gtg gcc tag gtt ggc ctg gct tac tca ggt gag gcc ttt aaa aga gag ctc aag cct
R V A - V G L A Y S G E A F K R E L K P
gcc ctg agc ttg gag aga ttt tcc tgc tag cct tga aga aac aaa cag ctg tga gtt ctg
A L S L E R F S C - P - R N K Q L - V L
cag ctt cac aga aat gaa ttt ggc cga caa tca tgt gag ctc cat tag aaa tgc acc cta
Q L H R N E F G R Q S C E L H - K C T L
agc tct ctc aac ctc tcc cat ccc cca cag cac cta ttt act gat tat ctg tac atg ata
S S L N L S H P P Q H L F T D Y L Y M I
gca ggg cag gaa cta gac tct cag ccc cct gag gac aga cct tgt ctg tca gtg cat ttc
A G Q E L D S Q P P E D R P C L S V H F
act gtc tgt ccc agc acc ttg cac aga gcc tgg cac ata tca aga aca cgg cac att ttt
T V C P S T L H R A W H I S R T R H I F
att gag aca gac tcc tca aag ctg gaa tct aaa acc aat ggc acc taa tct cag cca aaa
I E T D S S K L E S K T N G T - S Q P K
agg tta atg tga cac ctg tgc ttt tat tac cca ttt ctg cag atg gaa ggg ttg gtt gtt
R L M - H L C F Y Y P F L Q M E G L V V
tgt ggc tat ctc aag tga aat gca gaa tta gtg aat aaa att ttc atc tga att taa aga
C G Y L K - N A E L V N K I F I - I - R
aaa tct taa
K S -

2) LncRNA ENST00000647969.1

rs61871745

5'3' Frame 1

tct gac cca ggc acc ggt tat gaa gaa gac aag cag ctc tac aga ggg gcc tca aga aat
S D P G T G Y E E D K Q L Y R G A S R N
cct tcg aaa cag aaa tca aag tct tgc tgg aag ggt tct ggg cac cct gga ctg tcc aca
P S K Q K S K S C W K G S G H P G L S T
tgg gag gct gtt ggt gcg tgt gcc tac gtg tgt cca tca gtg gat gca cga aga agg aaa
W E A V G A C A Y V C P S V D A R R R K

```
tgc tgt cag ctg tct gca ctc cca gac ggg gtg atg gcc tgg cac caa cgg agc att acc  G/A=C/T, Thr > Ile
 C   C   Q   L   S   A   L   P   D   G   V   M   A   W   H   Q   R   S   I   T
ctg atg aat cat ggt cga ggt act ttt cca ctg ggc cag ccg cca cag ccc ctt ctc cat
 L   M   N   H   G   R   G   T   F   P   L   G   Q   P   P   Q   P   L   L   H
cct tcg gag agc tct gtc acc tcg cct tgt agc act tgt gtc tct ccc act ggc ctc cac
 P   S   E   S   S   V   T   S   P   C   S   T   C   V   S   P   T   G   L   H
ttc ttg tgg gca ctt tct gga tgc cca ggc cta atc cag cgc tgg cac tca gtt aat tct
 F   L   W   A   L   S   G   C   P   G   L   I   Q   R   W   H   S   V   N   S
ctc ttc ctt taa agg gaa caa tta cca cct tga gga agg agg cct gaa agc taa gtc ttt
 L   F   L   -   R   E   Q   L   P   P   -   G   R   R   P   E   S   -   V   F
gct att gca tcc gcc ttc ttc tca cga aga cct gca gct tga aag gag ggg agg gag aaa
 A   I   A   S   A   F   F   S   R   R   P   A   A   -   K   E   G   R   E   K
tag ccc taa gct cca gga aga cca
 -   P   -   A   P   G   R   P
```

5'3' Frame 2

```
tctg acc cag gca ccg gtt atg aag aag aca agc agc tct aca gag ggg cct caa gaa atc
  L   T   Q   A   P   V   M   K   K   T   S   S   S   T   E   G   P   Q   E   I
ctt cga aac aga aat caa agt ctt gct gga agg gtt ctg ggc acc ctg gac tgt cca cat
 L   R   N   R   N   Q   S   L   A   G   R   V   L   G   T   L   D   C   P   H
ggg agg ctg ttg gtg cgt gtg cct acg tgt gtc cat cag tgg atg cac gaa gaa gga aat
 G   R   L   L   V   R   V   P   T   C   V   H   Q   W   M   H   E   E   G   N
gct gtc agc tgt ctg cac tcc cag acg ggg tga tgg cct ggc acc aac gga gca tta ccc
 A   V   S   C   L   H   S   Q   T   G   -   W   P   G   T   N   G   A   L   P
tga tga atc atg gtc gag gta ctt ttc cac tgg gcc agc cgc cac agc ccc ttc tcc atc
 -   -   I   M   V   E   V   L   F   H   W   A   S   R   H   S   P   F   S   I
ctt cgg aga gct ctg tca cct cgc ctt gta gca ctt gtg tct ctc cca ctg gcc tcc act
 L   R   R   A   L   S   P   R   L   V   A   L   V   S   L   P   L   A   S   T
tct tgt ggg cac ttt ctg gat gcc cag gcc taa tcc agc gct ggc act cag tta att ctc
 S   C   G   H   F   L   D   A   Q   A   -   S   S   A   G   T   Q   L   I   L
tct tcc ttt aaa ggg aac aat tac cac ctt gag gaa gga ggc ctg aaa gct aag tct ttg
 S   S   F   K   G   N   N   Y   H   L   E   E   G   G   L   K   A   K   S   L
cta ttg cat ccg cct tct tct cac gaa gac ctg cag ctt gaa agg agg gga ggg aga aat
 L   L   H   P   P   S   S   H   E   D   L   Q   L   E   R   R   G   G   R   N
agc cct aag ctc cag gaa gac caa
 S   P   K   L   Q   E   D   Q
```

5'3' Frame 3

```
tctga ccc agg cac cgg tta tga aga aga caa gca gct cta cag agg ggc ctc aag aaa tcc
   -   P   R   H   R   L   -   R   R   Q   A   A   L   Q   R   G   L   K   K   S
ttc gaa aca gaa atc aaa gtc ttg ctg gaa ggg ttc tgg gca ccc tgg act gtc cac atg
 F   E   T   E   I   K   V   L   L   E   G   F   W   A   P   W   T   V   H   M
gga ggc tgt tgg tgc gtg tgc cta cgt gtg tcc atc agt gga tgc acg aag aag gaa atg
 G   G   C   W   C   V   C   L   R   V   S   I   S   G   C   T   K   K   E   M
ctg tca gct gtc tgc act ccc aga cgg ggt gat ggc ctg gca cca acg gag cat tac cct synonymous mutation
 L   S   A   V   C   T   P   R   R   G   D   G   L   A   P   T   E   H   Y   P
gat gaa tca tgg tcg agg tac ttt tcc act ggg cca gcc gcc aca gcc cct tct cca tcc
 D   E   S   W   S   R   Y   F   S   T   G   P   A   A   T   A   P   S   P   S
ttc gga gag ctc tgt cac ctc gcc ttg tag cac ttg tgt ctc tcc cac tgg cct cca ctt
 F   G   E   L   C   H   L   A   L   -   H   L   C   L   S   H   W   P   P   L
ctt gtg ggc act ttc tgg atg ccc agg cct aat cca gcg ctg gca ctc agt taa ttc tct
 L   V   G   T   F   W   M   P   R   P   N   P   A   L   A   L   S   -   F   S
ctt cct tta aag gga aca att acc acc ttg agg aag gag gcc tga aag cta agt ctt tgc
 L   P   L   K   G   T   I   T   T   L   R   K   E   A   -   K   L   S   L   C
tat tgc atc cgc ctt ctt ctc acg aag acc tgc agc ttg aaa gga ggg gag gga gaa ata
 Y   C   I   R   L   L   L   T   K   T   C   S   L   K   G   G   E   G   E   I
gcc cta agc tcc agg aag acc aag
 A   L   S   S   R   K   T   K
```

3) LncRNA ENST00000811416.1

rs61871745

5'3' Frame 1

```
gct ggt ggc ttc atc agg aag gga gtc agc agg ggg agg act ggg gga cca agc ggt ccc
 A   G   G   F   I   R   K   G   V   S   R   G   R   T   G   G   P   S   G   P
taa gaa gca gaa ttt gca ata ccc aag gtt tta gaa aga gca acc tgg cag ggc cac tgc
 -   E   A   E   F   A   I   P   K   V   L   E   R   A   T   W   Q   G   H   C
aca atg aga tca cag act cac ctc cca gct gtg agt gca gct cac ccc acc tgc tct gac
 T   M   R   S   Q   T   H   L   P   A   V   S   A   A   H   P   T   C   S   D
cca ggc acc ggt tat gaa gaa gac aag cag ctc tac aga ggg gcc tca aga aat cct tcg
 P   G   T   G   Y   E   E   D   K   Q   L   Y   R   G   A   S   R   N   P   S
aaa cag aaa tca aag tct tgc tgg aag ggt tct ggg cac cct gga ctg tcc aca tgg gag
 K   Q   K   S   K   S   C   W   K   G   S   G   H   P   G   L   S   T   W   E
gct gtt ggt gcg tgt gcc tac gtg tgt cca tca gtg gat gca cga aga agg aaa tga att
 A   V   G   A   C   A   Y   V   C   P   S   V   D   A   R   R   R   K   -   I
```

gat tcc att ctt gat gtg act ttc agc tgt cag ctg tct gca ctc cca gac ggg gtg atg
D S I L D V T F S C Q L S A L P D G V **M**
gcc tgg cac caa cgg agc att acc ctg atg aat cat ggt cga ggg aac aat tac cac ctt G/A=C/T, Thr to Ile
A W H Q R S I T L **M** N H G R G N N Y H L
gag gaa gga ggc ctg aaa gct aag tct ttg cta ttg cat ccg cct tct tct cac gaa gac
E E G G L K A K S L L L H P P S S H E D
ctg cag ctt gaa agg agg gga ggg aga aat agc cct aag ctc cag gaa gac caa gcc cag
L Q L E R R G G R N S P K L Q E D Q A Q
cct ctg tga tga ttc cga gga aat tca gga aat cct tgc taa tgg cca aac ttc ccg ggt
P L - - **F** R G N S G N P C - **W** P N F P G
ttg aga tgc aaa agg aaa tca gat cgc agg gta atg ctt tta cac taa gca aga aat tgc
L R C K R K S D R R V **M** L L H - **A** R N C
agg ata taa agc tgt gca gac cat tgc ctc tga tta aga aa
R I - **S** C A D H C L - **L** R

5'3' Frame 2

gctg gtg gct tca tca gga agg gag tca gca ggg gga gga ctg ggg gac caa gcg gtc cct
L V A S S G R E S A G G G L G D Q A V P
aag aag cag aat ttg caa tac cca agg ttt tag aaa gag caa cct ggc agg gcc act gca
K K Q N L Q Y P R F - **K** E Q P G R A T A
caa tga gat cac aga ctc acc tcc cag ctg tga gtg cag ctc acc cca cct gct ctg acc
Q - **D** H R L T S Q L - **V** Q L T P P A L T
cag gca ccg gtt atg aag aag aca agc agc tct aca gag ggg cct caa gaa atc ctt cga
Q A P V **M** K K T S S S T E G P Q E I L R
aac aga aat caa agt ctt gct gga agg gtt ctg ggc acc ctg gac tgt cca cat ggg agg
N R N Q S L A G R V L G T L D C P H G R
ctg ttg gtg cgt gtg cct acg tgt gtc cat cag tgg atg cac gaa gaa gga aat gaa ttg
L L V R V P T C V H Q W **M** H E E G N E L
att cca ttc ttg atg tga ctt tca gct gtc agc tgt ctg cac tcc cag acg ggg tga tgg
I P F L **M** - **L** S A V S C L H S Q T G - **W**
cct ggc acc aac gga gca tta ccc tga tga atc atg gtc gag gga aca att acc acc ttg
P G T N G A L P - - **I** **M** V E G T I T T L
agg aag gag gcc tga aag cta agt ctt tgc tat tgc atc cgc ctt ctt ctc acg aag acc
R K E A - **K** L S L C Y C I R L L L T K T
tgc agc ttg aaa gga ggg gag gga gaa ata gcc cta agc tcc agg aag acc aag ccc agc
C S L K G G E G E I A L S S R K T K P S
ctc tgt gat gat tcc gag gaa att cag gaa atc ctt gct aat ggc caa act tcc cgg gtt
L C D D S E E I Q E I L A N G Q T S R V
tga gat gca aaa gga aat cag atc gca ggg taa tgc ttt tac act aag caa gaa att gca
- **D** A K G N Q I A G - **C** F Y T K Q E I A
gga tat aaa gct gtg cag acc att gcc tct gat taa gaa a
G Y K A V Q T I A S D - **E**

5'3' Frame 3

gctgg tgg ctt cat cag gaa ggg agt cag cag ggg gag gac tgg ggg acc aag cgg tcc cta
W W L H Q E G S Q Q G E D W G T K R S L
aga agc aga att tgc aat acc caa ggt ttt aga aag agc aac ctg gca ggg cca ctg cac
R S R I C N T Q G F R K S N L A G P L H
aat gag atc aca gac tca cct ccc agc tgt gag tgc agc tca ccc cac ctg ctc tga ccc
N E I T D S P P S C E C S S P H L L - **P**
agg cac cgg tta tga aga aga caa gca gct cta cag agg ggc ctc aag aaa tcc ttc gaa
R H R L - **R** R Q A A L Q R G L K K S F E
aca gaa atc aaa gtc ttg ctg gaa ggg ttc tgg gca ccc tgg act gtc cac atg gga ggc
T E I K V L L E G F W A P W T V H **M** G G
tgt tgg tgc gtg tgc cta cgt gtg tcc atc agt gga tgc acg aag aag gaa atg aat tga
C W C V C L R V S I S G C T K K E **M** N -
ttc cat tct tga tgt gac ttt cag ctg tca gct gtc tgc act ccc aga cgg ggt gat ggc
F H S - **C** D F Q L S A V C T P R R G D G
ctg gca cca acg gag cat tac cct gat gaa tca tgg tcg agg gaa caa tta cca cct tga
L A P T E H Y P D E S W S R E Q L P P -
gga agg agg cct gaa agc taa gtc ttt gct att gca tcc gcc ttc ttc tca cga aga cct
G R R P E S - **V** F A I A S A F F S R R P
gca gct tga aag gag ggg agg gag aaa tag ccc taa gct cca gga aga cca agc cca gcc
A A - **K** E G R E K - **P** - **A** P G R P S P A
tct gtg atg att ccg agg aaa ttc agg aaa tcc ttg cta atg gcc aaa ctt ccc ggg ttt
S V **M** I P R K F R K S L L **M** A K L P G F
gag atg caa aag gaa atc aga tcg cag ggt aat gct ttt aca cta agc aag aaa ttg cag
E **M** Q K E I R S Q G N A F T L S K K L Q
gat ata aag ctg tgc aga cca ttg cct ctg att aag aaa
D I K L C R P L P L I K K
